# Iron in Friedreich Ataxia: A Central Role in the Pathophysiology or an Epiphenomenon?

**DOI:** 10.3390/ph11030089

**Published:** 2018-09-19

**Authors:** David Alsina, Rosa Purroy, Joaquim Ros, Jordi Tamarit

**Affiliations:** Departament de Ciències Mèdiques Bàsiques, IRBLleida, Universitat de Lleida, 25198 Lleida, Spain; david.alsina@cmb.udl.cat (D.A.); rosa.purroy@irblleida.udl.cat (R.P.); joaquim.ros@cmb.udl.cat (J.R.)

**Keywords:** Iron-sulfur, Friedreich Ataxia, Oxidative stress, Iron chelators

## Abstract

Friedreich ataxia is a neurodegenerative disease with an autosomal recessive inheritance. In most patients, the disease is caused by the presence of trinucleotide GAA expansions in the first intron of the frataxin gene. These expansions cause the decreased expression of this mitochondrial protein. Many evidences indicate that frataxin deficiency causes the deregulation of cellular iron homeostasis. In this review, we will discuss several hypotheses proposed for frataxin function, their caveats, and how they could provide an explanation for the deregulation of iron homeostasis found in frataxin-deficient cells. We will also focus on the potential mechanisms causing cellular dysfunction in Friedreich Ataxia and on the potential use of the iron chelator deferiprone as a therapeutic agent for this disease.

## 1. The Disease

Friedreich’s Ataxia (FRDA) is a neurodegenerative disease described at the end of the 19th century by the German physician Nikolaus Friedreich from whom acquired the name. Friedreich observed in a group of patients that during the puberty a characteristic symptomatology began to manifest, specifically: ataxia, dysarthria, loss of sensitivity, muscle weakness, scoliosis, pes cavus, and heart symptoms. Later, a greater incidence of diabetes mellitus in patients than in the rest of the population was also reported [1]. It is considered a rare disease that follows a pattern of autosomal recessive inheritance. The frequency of carriers oscillates, depending on the area, between 1:50 and 1:100, while those affected by the disease are approximately 1:50,000, which makes this disease the most common form of hereditary ataxia [2].

The mutation responsible for the disease is an expansion of GAA trinucleotides in the first intron of the FXNor X25 gene (located in chromosome 9), which codes for the mitochondrial protein frataxin [3]. This expansion of triplets, which in patients can reach up to more than 1000 copies, results in a marked decrease in protein frataxin levels (below 5%–30% of normal levels) [4]. The number of GAA expansions has an inversely proportional relation to the age at which the first symptoms of the disease begin to manifest and it also determines their severity [5,6,7]. Besides GAA expansions, epigenetic modifications might also contribute to the variability in the onset and disease progression. Sarsero and collaborators reported differences in DNA methylation patterns between patients upstream and downstream the GAA expansion. Such differences caused variations in frataxin gene expression [8]. Finally, a small percentage of patients, around 4%, are compound heterozygous for a GAA expansion and a frataxin (FXN) point mutation or deletion [9].

## 2. Frataxin, an Ancestral Conserved Protein

**Figure 1:**
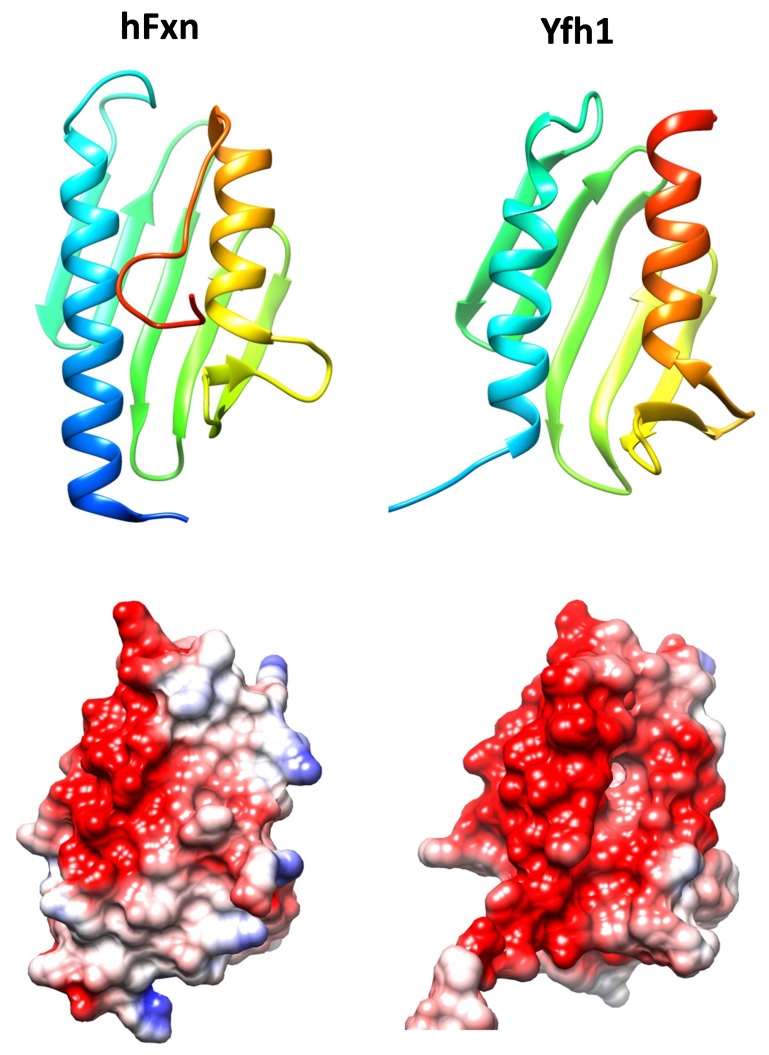
Structures of human frataxin (hFxn, pdb code 3s4m) and Yeast Frataxin Homologue 1 (Yfh1, pdb code 2fql). Top, ribbons representations showing the conserved alpha-beta-alpha structure. Structures are colored according to sequence, from dark blue (N-terminal) to red (C-terminal). In human frataxin the C-terminal region folds over the hydrophobic cavity formed between both alpha helices. Below, coulumbic surface coloring of the same structures. The red color indicates the presence of a marked acidic ridge, which may be involved in iron binding. Molecular graphics and analyses were performed with the UCSF Chimera package [11].

Frataxin is a highly conserved protein throughout evolution and homologues can be found in most species. Its structure is formed by two helix alpha joined by a series of antiparallel beta sheets and is highly conserved (Figure 1). Despite the high degree of conservation in the three-dimensional structure, the stability of this protein varies significantly between species. One of the factors that are responsible for the differences in the stability of the protein is the C-terminal endpoint. While in Yfh1 (the yeast’s homologue), this region is virtually nonexistent, in the human protein this fragment is found inserted between the two alpha helixes. This protects the hydrophobic nucleus of the protein and increases its stability [10].

Frataxin is a mitochondrial protein, and as such, it has a signal peptide at its N-terminal end. It was soon identified, both for Yfh1 and for mouse frataxin, that Mitochondrial Processing Peptidase interacted with frataxin and was responsible for its processing [12,13]. This processing involves two sequential cleavages, which first produce an intermediate form of frataxin and subsequently the mature form. For human frataxin, the cleavage positions described are between amino acids 41–42 (intermediate form) and between amino acids 80–81 (mature form), although other less abundant forms that correspond to alternative cleavage sites (for instance, positions 55–56) have also been identified [14,15]. In these works, it was shown that the mature form of frataxin corresponds to FXN^81–210^. This form is fully active and capable of improving the survival and phenotypes of frataxin deficient cells. Despite the demonstration that the FXN^81–210^ form is the dominant form and that it is active, there is some debate over the role that could be developed by the intermediate forms, as these forms can also be detected in certain tissues (although at low levels) [16]. It was shown that processing of the intermediate form was slower than that of the precursor form, which suggested that this could be a mechanism for controlling the levels of the different frataxin forms [17]. More recently, it has been suggested that the different forms of frataxin might play different roles. This point will be discussed in the next section. In addition to post-translational processing, alternative splicing mechanisms have also been observed that may generate different isoforms of frataxin. Work by Xia and collaborators suggested that these alternative forms could be tissue-specific and could have different functions and locations [18]. However, there are not additional evidences about the nature and relevance of these alternative forms.

## 3. Frataxin Function

Although very early after the discovery of frataxin as the gene that was responsible for Friedreich’s Ataxia, it was established that iron homeostasis was altered by frataxin deficiency, the specific function of this protein remains controversial. Over the years, different functions have been proposed for frataxin, most of them related to iron metabolism and the control of oxidative stress in mitochondria.

### 3.1. Frataxin, an Iron Binding and Storage Protein

It has been proposed that frataxin could work like a metalochaperone and iron storage protein. In several studies with purified protein, it has been observed that frataxin has the ability to interact with metal ions, but the coordination environment of these metal binding sites has not been properly defined. There is also uncertainty on the number of metal ions that are bound per frataxin monomer. It has been described that frataxin can bind divalent metal ions using a group of exposed acidic residues. These amino acids are located in a specific area of the protein forming an acidic ridge, quite conserved, different from the canonical iron binding motifs where cysteine and/or histidine amino acids are usually found. This acidic zone results in a weak and non-specific electrostatic bonding of iron, which also allows the coordination of other divalent metals [19]. The estimated Kd for Fe^2+^ and Fe^3+^ of this region was calculated on the micromolar range [20]. Gentry and collaborators reported the potential presence of a high affinity iron binding site. They showed that three metal ions could be bound by each frataxin monomer, and that His86 was required for one of these binding sites. This residue is located in the disordered N-terminal tail and had not been previously reported to be involved in metal coordination. They calculated that this site would have an affinity for Fe^2+^ higher than the iron chelator ferrozine, while the remaining two sites would have lower affinities [21]. His86 is not included in most of the frataxin structures that are found in the protein data bank nor is conserved in yeast and bacterial homologues. More recently, while using NMR to investigate iron binding, it was also proposed that frataxin tightly binds a single Fe^2+^ but not Fe^3+^ [22].

Isaya and collaborators noticed that iron binding to yeast and bacterial frataxin promoted its oligomerization to complexes of high molecular weight (850–1100 kDa) [23,24,25]. These oligomeric forms resembled those that were formed by ferritin, the main protein responsible for iron storage in eukaryotes. Indeed, oligomeric frataxin was shown to use a ferrooxidation reaction to build a ferrihydrite mineral core inside the particles. Therefore it was proposed that frataxin could act as a mitochondrial ferritin. Although this function would be redundant in higher eukaryotes due to the presence of a mitochondrial ferritin, this hypothesis acquired some strength when subsequent studies demonstrated that the expression of mitochondrial ferritin in frataxin deficient yeast was able to partially recover some of the observed phenotypes. Specifically, the heterologous expression of mitochondrial ferritin partially prevented the accumulation of iron, the cells were more resistant to oxidizing agents and they partially recovered the activities of enzymes with iron-sulfur centers (which is a common described consequence of frataxin deficiency) [26]. Regarding human frataxin, it has been claimed that the mature form (FXN^81–210^) does not form aggregates [27] and that only the intermediate forms FXN^42–210^ and FXN^56–210^ would be assembled into larger structures. Based on this observation, it has been suggested that different frataxin proteoforms would perform different functions. The mature form would be monomeric and involved in iron binding and delivery to biochemical pathways requiring this metal, while FXN^56–210^ and FXN^42–210^ would be able to oligomerize and store iron. A caveat to these hypotheses is that the long frataxin isoforms are not observed in most tissues by western blot. Mass spectrometry data also suggests that these long frataxin isoforms may be present at very low concentrations: data collected in the PeptideAtlas repository indicates that the theoretically likely frataxin peptides between positions 42 and 81 have never been observed, while those from the FXN^81–210^ have been observed at least 400 times. (PeptideAtlas is a publicly accessible compendium of peptides identified in mass spectrometry proteomics experiments) [28]. Recently, it has been shown that FXN^81–210^ can also undergo oligomerization under certain conditions, although the stability of these oligomers would be lower than that of bacterial frataxin [29].

Nevertheless, there are other caveats on the ferritin-like hypothesis. It has been argued that physiological conditions of calcium and magnesium stabilize the monomeric frataxin form and consequently frataxin would not oligomerize in vivo [27]. It has also been shown that mitochondrial iron in yeast strains expressing different Yfh1 concentrations, presented nearly identical chemical and biochemical characteristics [30]. Another point to take into account is which could be the contribution of frataxin to mitochondrial iron storage or trafficking from a quantitative point of view. Most iron that is present in mitochondria is found at the active sites of proteins. Despite that, non-proteinaceous labile metal iron pools have also been detected within cells. These pools are thought to be involved in cellular trafficking, regulation, signaling, and/or storage of metal ions. Due to their lability (and their presence at low concentrations), their structure and functions are not completely understood. For instance, mitochondria contain 0.7–2 mM Fe, but the proportion of labile iron is not completely known, with estimates ranging from 1 to 100 μM [31,32]. These differences in the estimates may be due to real differences between the models or experimental conditions used, but also on the methodological approaches used to analyze this elusive iron pool. Also, the nature of these nonproteinaceous metal complexes is not known. Based on the abundance of GSH within the mitochondria it has been hypothesized that could be an FeII (GSH) adduct. Citrate, which is also present in the mitochondrial matrix at high concentrations, has also been considered as a potential ligand for non-proteinaceous iron complexes (reviewed in [32]). In yeast, by using Mössbauer spectroscopy, it has been shown that the proportion and nature of these labile iron pools may vary depending on the metabolic state of the cell. Respiring mitochondria where estimated to contain ∼15 μM labile non-heme high spin Fe^2+^, while this pool in fermenting mitochondria increased to ∼150 μM. The concentration of yeast frataxin in mitochondria has been estimated to be three orders of magnitude lower, around 35 nM [33]. Therefore, as monomeric frataxin has been claimed to bind three iron atoms, it cannot contribute significantly to store iron in a non-reactive easy deliverable form. However, it could play a role in iron trafficking as a temporary carrier or catalyzing its speciation between different forms. For instance, it could bind Fe^2+^ and promote its controlled oxidation to Fe^3+^, which would be then stored in the form of Fe^3+^-phosphate nanoparticles. Regarding oligomeric frataxin, the mineralization process would allow for this protein to bind much more iron atoms per subunit. The yeast 48 subunit oligomer can store ∼50–75 iron atoms per subunit in 1–2 nm cores [34]. This raises the iron potentially bound by frataxin up to the μM range, but still this amount may not be a significant contribution to the whole mitochondrial iron pool. In comparison, around 35% of mitochondrial iron in fermenting mitochondria may be stored in the form of Fe^3+^ nanoparticles [33]. That said, the contribution of mitochondrial ferritin to iron storage in mitochondria is also intriguing, as the concentration of this protein according to the PaxDb database is much lower than that of frataxin (PaxDB is a database that contains protein abundance information across organisms and tissues) [35].

### 3.2. Frataxin in the Biosynthesis of Iron Containing Proteins

It has also been proposed that frataxin could participate in the biosynthesis of both heme groups and of iron-sulfur centers. This hypothesis has several fundamentals: (a) frataxin deficiency leads to a loss in proteins which contain iron-sulfur centers or heme groups; (b) frataxin has the ability to bind iron atoms; and, (c) several studies have shown the ability of frataxin to interact with proteins that areinvolved in heme or iron-sulfur biosynthesis.

#### 3.2.1. Biosynthesis of Heme Groups

The incorporation of the iron atom into protoporphyrin IX is the last step in the biosynthesis of heme groups. This step is catalyzed by ferrochelatase, but it is not known how iron is provided to this enzyme. In in vitro studies, it was shown that there was a physical interaction between frataxin and ferrochelatase with a 1 to 2 stoichiometry, which seems logical, since ferrochelatase functions as a dimer. In addition, this interaction resulted in the formation of heme groups [36]. A study in which NMR spectroscopy was used to analyze the binding between both of the proteins suggested that ferrochelatase interacted with frataxin in a manner that included its iron-binding interface [37]. More recently, Söderberg and collaborators presented a model of the interaction of trimeric Yfh1 (yeast frataxin) and ferrochelatase in which one of the subunits of the Yfh1 trimer interacted with one subunit of the ferrochelatase dimer, whereas another trimer subunit was positioned for iron delivery [38]. These results support the hypothesis of frataxin acting as an iron donor in the biosynthesis of heme groups. However, heme deficiency is not always observed in frataxin-deficient cells and anemia has not been shown to be a symptom of FRDA. Indeed, no alterations where observed in heme synthesis in erythroid progenitor stem cells that were obtained from FRDA patients [39]. Moreover, experiments using either conditional frataxin-deficient T-Rex-293 cells or yeasts have shown that heme deficiency is a late consequence of frataxin deficiency [40,41]. These observations suggest that heme deficiency may be an epiphenomenon observed in certain frataxin-deficient cells which could be caused by poor iron availability or by metabolic remodeling. In this regard, we have shown in frataxin-deficient yeast that heme deficiency could be caused by the induction of Cth2, a protein induced in response to iron limitation, which promotes the degradation of mRNAs from iron-containing proteins [42].

#### 3.2.2. Biosynthesis of Iron-Sulfur Centers

In addition to interacting with ferrochelatase, frataxin also interacts with the proteins that form the central biosynthesis machinery of iron-sulfur centers: IscU (Isu1 in yeast), Nfs1, and Isd11 [43,44]. Several authors have shown that this interaction facilitates the formation of an iron-sulfur center into IscU, the scaffold protein where these cofactors are first assembled. It was initially suggested that frataxin would act as an iron donor in the biosynthesis of these centers [45]. More recently, it has been suggested that frataxin would participate in the biosynthesis of iron-sulfur centers as an allosteric regulator and not as an iron donor. In works that were carried out in vitro with the CyaY protein (bacterial homologue of frataxin), it was shown that this protein had an inhibitory effect on the production of iron-sulfur centers. As this effect was increased in response to iron concentration, the authors suggested that frataxin could adapt the production of iron-sulfur to the number of final acceptor proteins [46]. Surprisingly, studies with human proteins demonstrated that eukaryotic frataxins would have a contrary effect. In this case, they stimulated the production of iron-sulfur centers by favoring the desulfurase activity of Nfs1 [47,48,49].

Today, despite the intense debate that is generated around the function or functions of frataxin, this non-essential activity in the metabolism of iron as an allosteric regulator of the biosynthesis of iron-sulfur centers has strong support from in vitro biochemical data and is the most accepted hypothesis. A detailed explanation of this complex biochemical process can be found in recent reviews [50,51]. However, this hypothesis also presents caveats when exposed to in vivo biological data. Remarkably, iron-sulfur cluster deficiency is not observed in several models of frataxin deficiency, such as flies [52], patient fibroblasts [53], or rat cardiac myocytes [54]. Moreover, detailed analysis of the cellular events that are caused by frataxin deficiency in yeast, have shown that iron-sulfur deficiency is an epiphenomenon that is caused by a metabolic remodeling program activated in response to disrupted iron homeostasis [41,55]. These observations question the role of frataxin in iron-sulfur biogenesis or at least suggest the possibility of additional functions for frataxin beyond iron-sulfur biogenesis. From these observations, it also becomes obvious that frataxin is not essential for iron-sulfur biogenesis.

### 3.3. Control of Oxidative Stress and the Generation of Ros

One of the phenotypes most consistently observed in frataxin-deficient cells is sensitivity to oxidizing agents [56,57]. Some authors have linked such sensitivity to oxidative stress to impaired biosynthesis of iron-sulfur centers. This hypothesis suggests that a vicious cycle would be created in which the deficient formation of iron-sulfur clusters would increase mitochondrial free iron that would increase the production of reactive oxygen species (ROS) through Fenton reaction. Then, ROS would further damage iron-sulfur clusters and promote the formation of more free iron. In fact, increased presence of labile iron has been reported in frataxin deficient yeast [58], T-Rex-293 cells [40] and in a mouse model of hepatic FXN deficiency [59]. However, no differences where observed between the mitochondrial iron pools from human lymphoblasts and fibroblasts that were obtained from either controls or FRDA patients [31]. Nevertheless, some observations suggest that oxidative stress could be the cause (and not the consequence) of iron-sulfur deficiency. For instance, in fibroblasts or lymphocytes from patients [53], or in frataxin-deficient cardiac myocytes [54], oxidative stress could be observed while the activities of iron-sulfur proteins remained unaltered. Moreover, there are evidences that iron-sulfur deficiency can be modulated by oxygen concentration or antioxidant treatment. In this regard, frataxin is not required for iron-sulfur biogenesis in yeasts grown at low oxygen tensions [60,61]. In some fly models, iron-sulfur deficiency is only observed under hyperoxic conditions [52], while in other models it can be prevented by antioxidants [62].

Which could be the origin of oxidative stress? Since frataxin has the ability to bind iron, a redox active metal, and oxidize it to Fe^3+^, it has been proposed that frataxin could prevent oxidative stress by limiting the presence of free Fe^2+^ through its binding and the subsequent controlled oxidation to Fe^3+^. This reaction would prevent the formation of reactive oxygen species through the reaction of Fe^2+^ with oxygen [63,64]. Therefore, the vicious cycle would have its origin in free iron than would then generate oxidative stress that would damage iron-sulfur clusters and generate more free iron. Another potential source of reactive oxygen species in frataxin-deficient cells could be the OXPHOS system. In this regard, an interaction was described between frataxin and mitochondrial electron transport chain proteins [65]. Also, decreased activity of the mitochondrial electron transport chain has been observed in several biological models of frataxin deficiency [53,66,67]. Any alteration in the OXPHOS system that was caused by frataxin deficiency could increase electron leakage and thus generate ROS [68].

Rustin and collaborators observed that frataxin-deficient cells could not properly activate the NRF2 signaling pathway in response to oxidative damage and in consequence they had a deficient response to oxidative insults and hypersensitivity to oxidative stress. They hypothesized that this impairment was related to actin remodeling [69]. This phenomenon has also been described in frataxin-deficient motor neurons [70], and in the frataxin-deficient YG8R mouse model where transcriptomic analysis showed a downregulation of NRF2-dependent antioxidant enzymes [71].

## 4. Evidences of Iron Accumulation and Its Relation to Pathophysiology in FRDA

Iron accumulation in a frataxin deficient cell model was first described in yeast *yfh1* mutants [72]. This early observation has been subsequently confirmed by several other researchers. Using Mössbauer spectroscopic analysis, Dancis and collaborators showed that in *Dyfh1* mitochondria iron was present as amorphous nano-particles of ferric phosphate [73]. Iron accumulation is caused by increased iron uptake due to activation of the iron sensor Aft1 [58], but the mechanisms leading to such activation are not completely understood. It has been assumed that it would be caused by iron-sulfur cluster loss, as Aft1 is known to be regulated by the presence of these cofactors. However, previous research from our group using conditional Yfh1 mutants provided two observations that challenged this hypothesis: (i) activation of Aft1 was observed earlier than iron-sulfur loss [41]; and, (ii) loss of iron-sulfur containing proteins in Yfh1 deficient yeasts was not observed in *cth2* cells. Therefore iron-sulfur loss was an epiphenomenon mainly caused by Cth2, which is an Aft1 target that binds to mRNAs from iron-containing proteins and promotes its degradation [42]. Moreover, we have also observed that nitric oxide can prevent Aft1 activation in Yfh1-deficient cells, but not in cells that are deficient in iron-sulfur biogenesis [74]. This observation also indicates that in Yfh1 deficient yeast Aft1 may be activated by a mechanism different than iron-sulfur cluster deficiency. Besides yeast, iron deposits or accumulation have also been clearly observed in frataxin deficient flies [75,76] and in cardiac muscles from frataxin deficient mice [77] and FRDA patients [78]. Iron in the heart from cardiac KO conditional mouse (the MCK mutant) was found in mitochondrial aggregates 100–400 nm in diameter, markedly different from those observed for mammalian ferritin. Energy-dispersive X-ray analysis showed that, in addition to iron, phosphorus and sulfur were present in these aggregates. Mössbauer spectra also confirmed that these aggregates where different than mammalian ferritin. The absorption profile observed was consistent with paramagnetic high-spin Fe(III) [79]. These observations are consistent with those that were obtained in frataxin-deficient yeast, and suggest that iron could be in the form of ferric-phosphate nanoparticles in both models. In other tissues or mammalian cell types, iron accumulation is not consistently observed. For instance, in fibroblasts or lymphoblasts from patients, there are no evidences of iron accumulation [31], while some authors have observed it in the nervous system [80]. Changes in the iron-responsive proteins, ferritin, divalent metal transporter 1 (DMT1), transferrin receptor 1 (TfR1), and ferroportin have been reported in the dentate nucleus of affected individuals [81]. Similar to yeast, iron deregulation in mammals might be caused by Iron-responding protein 1 (IRP1) activation [82,83], but the mechanisms causing this activation are not completely understood. It could be caused by deficiencies in iron supply to mitochondrial iron-dependent pathways that would activate the mechanisms of response to iron deficiency [59]. Frataxin has also been shown to interact with IRP1 and modulate the switch between its aconitase and RNA-binding forms. This function would be carried on by a cytosolic form of frataxin [84]. However, some authors are skeptical about the existence of an extra mitochondrial form of frataxin, and therefore question the physiological relevance of the observed interaction between IRP1 and frataxin.

As indicated above, many evidences support that frataxin deficiency causes a dysregulation in iron homeostasis, and it has also been shown in several models that the modulation of iron homeostasis ameliorates several phenotypes caused by frataxin deficiency [74,85]. However, the contribution of iron accumulation to the pathophysiology of FRDA has not been clearly determined. In this regard, several hypotheses have been formulated. It has been proposed that iron accumulation would be toxic and could be contributing to the formation of reactive oxygen species through the Fenton reaction. Iron overload could be also inducing the synthesis of sphingolipids, which, through the Pdk1/Mef2 pathway, would trigger neurodegeneration [76,80]. Iron toxicity could be also related to the formation of iron-phosphate aggregates that would compromise phosphate availability [86]. Nevertheless, it has also been suggested that iron accumulation would not be toxic per se, and that pathological consequences of frataxin deficiency would be mostly related to deficient iron supply to iron-dependent proteins. In this regard, it has been shown that IRP1 activation has a protective effect in a mouse model of hepatic FXN deficiency, as it contributes to sustain mitochondrial iron needs and mitochondrial function in these mice [59]. It has also been observed that dietary iron supplementation limits cardiac hypertrophy in MCK mutant mice [79].

These contradictory observations suggest that the pathological mechanisms could be more complex and specific for different models and tissues. For instance, in yeast, we have observed that activation of Aft1 causes the overexpression of Cth2, an mRNA binding protein that downregulates the expression of most iron-binding proteins that are required for aerobic growth. Thus, the activation of such pathway has a strong contribution to the alterations observed in yeast [42]. Beyond yeast, there are several evidences that frataxin deficiency may be causing perturbations in signaling pathways that could contribute to pathology. For instance, as mentioned before, neurodegeneration has been linked to the activation of the Pdk1/Mef2 pathway [76,80]. Cardiac hypertrophy could be related to the activation of the NFAT/calcineurin pathway, which has been observed in rat frataxin deficient cardiac myocytes [87]. Therefore, pathophysiology could be related to the pathways activated in different cells and tissues in response to the perturbations caused by frataxin deficiency.

Besides iron, some authors have reported deregulation of the homeostasis of other metals as a consequence of frataxin deficiency. In frataxin deficient yeast, we observed a decrease in manganese content and limited copper availability [58,88]. Subsequent studies using a conditional frataxin mutant indicated that manganese deficiency was caused by downregulation of Smf2, a Mn transporter that was degraded in response to iron accumulation [41]. In frataxin deficient flies it was found that the levels of zinc, copper, and manganese were increased, and that copper and zinc chelation improved the impaired motor performance of these flies [89]. In Dorsal Root Ganglia from FRDA patients, zinc and iron related proteins displayed major shifts in their cellular localization [90]. Alterations in calcium homeostasis have also been reported in several models of FRDA [87,91]. These alterations are mostly considered to be consequences of the deregulation of iron homeostasis, which may impact other metals. Nevertheless, frataxin is known to have also the capacity to chelate metals that are different than iron, such as manganese or copper [92]. The biological significance of these interactions has not been explored yet.

## 5. Targeting Iron as a Therapeutic Approach in FRDA

There is currently no cure for FRDA but several therapeutic approaches are being investigated. Some drugs have already entered clinical trials. Briefly, therapeutic approaches can be divided into compounds that improve mitochondrial function and reduce oxidative stress, drugs that modulate the altered metabolic pathways, and strategies to increase the expression or content of frataxin, either by promoting its expression, by supplying it through gene therapy (reviewed in [93]) or by protein replacement strategies [94].

Due to the alterations that were observed in iron homeostasis in different models and patients of FRDA, the use of iron chelators as a treatment to eliminate the excess iron accumulating in mitochondria was proposed many years ago. Deferoxamine was not considered to be a suitable iron chelator for depleting the intracellular iron deposits found in FRDA, as it does not cross the blood brain barrier and poorly penetrates biological membranes. It also has a high affinity for iron, which could compromise iron availability. The proposed alternative was deferiprone, an orally administered, lipidsoluble iron chelator that had been previously used to treat iron overload in polytransfused individuals with hemoglobinopathies. This compound can easily cross the blood–brain barrier and cellular membranes and therefore reach intracellular (or mitochondrial) iron deposits. In addition, since its affinity for iron is lower than that of transferrin, it has been shown that it can redistribute iron from intracellular iron deposits to this protein [95]. Indeed, the cellular properties that are affected by frataxin deficiency in HEK-293 cells were corrected by deferiprone treatment [96].

Several clinical trials have been performed with deferiprone in FRDA patients. In summary, these studies suggested that low doses of deferiprone would be beneficial on cardiac parameters. Higher doses of the drug worsened the condition and could result in agranulocytosis (reviewed in [97]). We can speculate that this dose dependent effect could be a consequence of different pathological mechanisms that are exerted by frataxin deficiency. Some of them would be caused by iron accumulation, while others would be caused by deficient iron availability. Therefore, low doses of the chelator would partially prevent the toxic effects that are caused by iron accumulation, while higher doses of deferiprone would compromise iron availability, and therefore worsen those pathological conditions caused by inefficient iron availability. Nevertheless, it has been proposed that deferiprone at low doses could be combined with other drugs. A pilot study with five FRDA patients suggested that combined therapy of deferiprone and idebenone (a Q10 analogue) was relatively safe and it could provide some benefits on neurological function and heart hypertrophy [98].

## 6. Concluding Remarks

**Figure 2:**
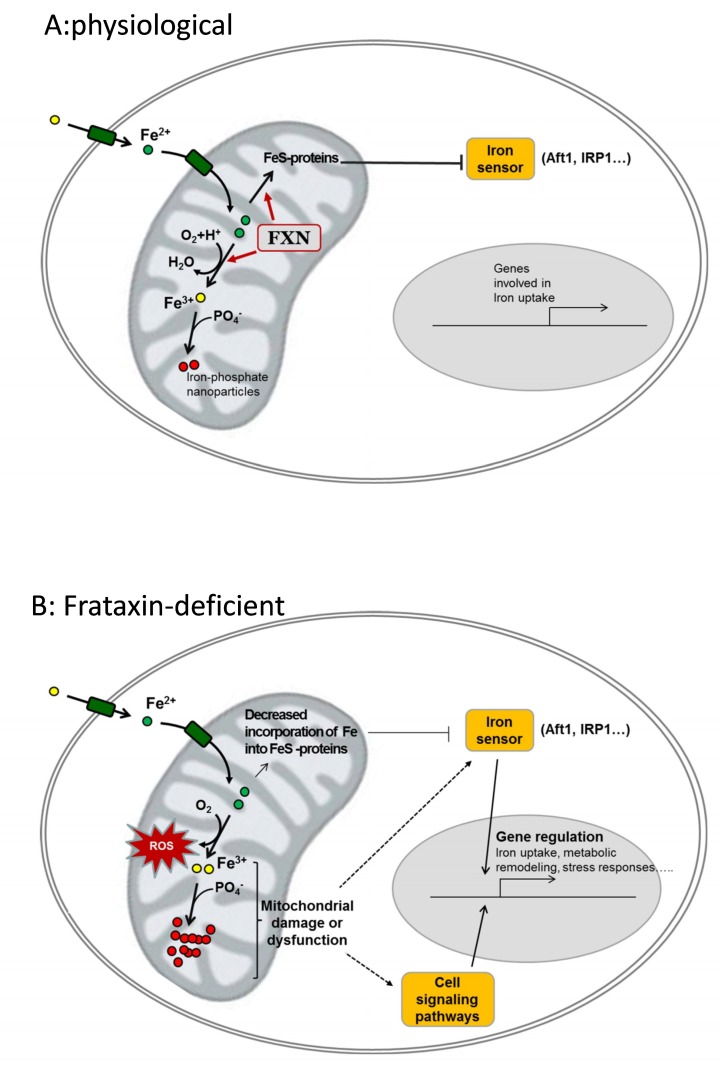
Potential contribution of frataxin to iron homeostasis and cellular consequences of its deficiency. (**A**), physiological: frataxin (FXN) binds Fe^2+^ and contributes to its controlled oxidation to Fe^3+^ and/or to incorporate it into Fe-containing proteins. These Fe-containing proteins (notably FeS proteins) keep the iron sensor inactive and genes involved in iron uptake are not expressed. Oxidized iron (Fe^3+^) is stored in the form of ferric-phosphate nanoparticles. (**B**), frataxin-deficient: loss of frataxin leads to decreased incorporation of iron into Fe-proteins and/or uncontrolled oxidation of Fe^2+^ by O_2_. Such events lead to reactive oxygen species (ROS) generation, decreased phosphate availability, and mitochondrial dysfunction. Iron sensors and other cell signaling pathways are activated and regulate the expression of genes involved in iron uptake and/or other cell-specific pathways involved on metabolic remodeling, hypertrophy or neurodegeneration.

Many evidences indicate that the lack of frataxin leads to alterations in iron cellular homeostasis. However, the precise mechanism(s) causing iron deregulation in frataxin-deficient cells are not completely understood. Several hypotheses have been formulated, but although most of them are well supported by in vitro data, all of them present caveats when exposed to biological data. In Figure 2, we have summarized two potential mechanisms which in our opinion could explain iron accumulation and oxidative stress: (1) the iron-sulfur hypothesis proposes that frataxin contributes to iron-sulfur biogenesis and its deficiency activates cellular iron sensors that would promote iron uptake; and, (2) the iron toxicity hypothesis assumes that frataxin would be involved in controlled iron ferrooxidation, and its deficiency would lead to ROS generation and the increased formation of ferric-phosphate nanoparticles. The iron-sulfur hypothesis is well supported by in vitro data, but its major caveat is the absence of iron-sulfur deficiency in many models of frataxin deficiency. On the other hand, the iron toxicity hypothesis does not provide a clear explanation for the activation of iron sensors.

Also, it is not clear the contribution of iron to FRDA pathology, which could be related either to iron accumulation or to limited iron availability. Indeed, iron homeostasis deregulation could be an epiphenomenon that is not linked to pathology. In fact, many evidences suggest that the mechanisms causing cellular dysfunction could be tissue or model specific. They could also be related to the signaling pathways activated in response to the alterations that are caused by frataxin deficiency. This complexity may explain the limited effects of iron chelators on clinical trials, as these compounds would only prevent certain pathological mechanisms in a limited number of tissues.
